# Co-creating a global shared research agenda on violence against women in low- and middle-income countries

**DOI:** 10.1186/s12961-024-01153-3

**Published:** 2024-06-24

**Authors:** Julienne Corboz, Elizabeth Dartnall, Chay Brown, Emma Fulu, Sarah Gordon, Mark Tomlinson

**Affiliations:** 1 Independent Consultant, Barcelona, Spain; 2 Sexual Violence Research Initiative, Pretoria, South Africa; 3 The Equality Institute, Melbourne, Australia; 4 Stellenbosch University, Stellenbosch, South Africa; 5 Queens University, Belfast, United Kingdom

**Keywords:** Violence against women, Intimate partner violence, Violence prevention interventions, Violence response interventions, Research priority setting, Research agenda, CHNRI method

## Abstract

**Background:**

Despite a large growth in evidence on violence against women (VAW) over the last 25 years, VAW persists, as do gaps in the field’s knowledge of how to prevent and respond to it. To ensure that research on VAW in low- and middle-income countries (LIMCs) is addressing the most significant gaps in knowledge, and to prioritise evidence needs to reduce VAW and better support victims/survivors, the Sexual Violence Research Initiative (SVRI) and Equality Institute (EQI) led a process of developing a global shared research agenda (GSRA) on VAW in LMICs.

**Methods:**

The GSRA was developed through a six-stage adaptation of the Child Health and Nutrition Research Initiative (CHNRI) method, which draws on the principle of the ‘wisdom of the crowd’. These steps included: a review of the literature on VAW in LMICs and development of domains; the generation of research questions within four domains by an Advisory Group; the consolidation of research questions; scoring of research questions by a Global Expert Group and the Advisory Group according to three criteria (applicability, effectiveness and equity); consultation and validation of the findings with the Advisory Group; and wide dissemination of the findings.

**Results:**

The highest ranked research questions in the GSRA pertain to the domain of Intervention research, with some highly ranked questions also pertaining to the domain of Understanding VAW in its multiple forms. Questions under the other two domains, Improving existing interventions, and Methodological and measurement gaps, were not prioritised as highly by experts. There was strong consistency in top ranked research questions according to experts’ characteristics, albeit with some important differences according to experts’ gender, occupation and geographical location.

**Conclusions:**

The GSRA findings suggest that currently the VAW field is shifting towards intervention research after several decades of building evidence on understanding VAW, including prevalence, drivers and impacts of violence. The findings also suggest a strong emphasis on under-served populations, and under-researched forms of VAW. Future priority setting exercises in LMICs that seek to decolonise knowledge should ensure that methodologies, and modalities of engagement, put diverse voices at the centre of engagement.

*Trial registration* Not applicable

## Background

The last twenty-five years has seen enormous growth of research on violence against women (VAW), particularly intimate partner violence (IPV) and non-partner sexual violence (NPSV). The addition of the domestic violence module to the Demographic and Health Survey in 1998 spearheaded the collection of national-level prevalence data on IPV and other forms of domestic violence across multiple country and regional contexts [1]. Although the domestic violence module is optional, by 2020, 65 countries had administered the module [2]. The World Health Organization (WHO) multi-country study on women’s health and domestic violence conducted between 2000 and 2003 was also pivotal in generating evidence on the global prevalence of VAW, with approximately one in three women found to have experienced physical or sexual violence in their lifetime, with the large majority having experienced violence from an intimate partner [3, 4]. The study also made an important contribution to the literature on factors associated with women’s experience of IPV [5]. More recent syntheses of evidence suggest that IPV and NPSV are particularly prevalent in low- or middle-income countries (LMICs) [6]. There is also growing evidence of the harmful impacts of VAW, including on physical, sexual and mental health outcomes [7–9].

The literature on interventions to respond to and prevent VAW has also expanded in the last few decades. Studies have ranged from research and evaluation on the effectiveness and impact of specific violence prevention or response interventions, to systematic and synthesis reviews to identify what works to prevent and respond to VAW [10–13]. Evidence reviews have also identified key gaps in the field, including rigorous evaluations of VAW response and prevention interventions being concentrated in high-income countries (HICs), with little evidence on how these interventions could be implemented or adapted in LMICs [14]. More recently, the What Works to Prevent Violence Against Women and Girls Global Programme (2014–2020), which involved the implementation and rigorous testing of 16 violence prevention interventions, generated important evidence on the most important elements of successful violence prevention interventions in South and Central Asia and Africa [15]. The evidence from the first phase of the ‘What Works’ programme confirmed that VAW is preventable. Building on this learning, phase two of the programme, which will be implemented over 7 years, focuses on scaling up successful prevention approaches and generating evidence on the effective and ethical scale of violence prevention interventions [16].

Despite this growth in evidence, VAW persists, as do gaps in the field’s knowledge of how to best prevent and respond to it, and how to scale up effective and impactful interventions, particularly in LMICs. There has also been a growing acknowledgement that in a field that has been historically dominated by Northern researchers and academics, advancing knowledge of VAW and how to address it must prioritise the decolonisation of knowledge and support Southern epistemologies and diverse, intersectional voices in the setting of research priorities [17, 18]. Indigenous scholars are overwhelmingly the architects of decolonising methodologies, and the experience of colonised Indigenous peoples is vital in these conversations, irrespective of whether they live in higher or lower income contexts.

To ensure that research on VAW in LMICs is addressing critical gaps in knowledge, and to prioritise evidence needs to reduce VAW and better support victims/survivors, the Sexual Violence Research Initiative (SVRI) and Equality Institute (EQI) in 2019 began a process of developing a global shared research agenda (GSRA) on VAW in LMICs. Central to this process was identifying and adapting an approach that was inclusive of the voices of multiple actors across the VAW prevention and response field and, in particular, would elevate the voices of actors in LMICs.

Several frameworks are available to guide research priority setting processes. These frameworks can be broadly grouped into two categories: a consensus-based approach, or a metrics-based approach. Consensus-based approaches develop priorities based on group consensus with a focus on acceptability of the exercise for the contributing group participants. In contrast, metrics-based approaches focus on metrics resulting in pooled individual rankings of research priorities that reduce the dominance of the voices of a few, powerful stakeholders [19].

The GSRA was developed through a six-stage adaptation of the Child Health and Nutrition Research Initiative (CHNRI) method. CHNRI is an example of a metrics-based approach [20]; however, our adaptation of the CHNRI method is an amalgamation of consensus- and metrics-based approaches. We significantly broadened the participation processes and simplified the metric aspects of the method to enable the active inclusion of voices that have historically been absent from priority-setting exercises.

The CHNRI method was developed to respond to a number of challenges and limitations in priority-setting exercises, a key one being that priorities tended to be set by a small group of academic researchers or experts, particularly those with power, with the views of other stakeholders rarely being included. Another important limitation was that priority setting exercises were not always based on a common set of criteria to guide decision making and were rarely open to external scrutiny [21]. To counter these limitations, the CHNRI method draws on the principle of the ‘wisdom of the crowd’ whereby research questions or options are: generated by experts in the field according to key domains of research; compiled and consolidated; and shared with the same group of experts for independent scoring according to pre-defined criteria [19, 22].

In line with an emphasis on decolonising knowledge and valuing all knowledge holders, the GSRA defined an ‘expert’ as encompassing diverse roles within the VAW prevention and response field, including researchers and practitioners.

## Methods

### Aim and purpose

The aim of the GSRA on VAW in LMICs was to establish key priority research questions for the VAW prevention and response field for the next five years, with an emphasis on equitable inclusion of multiple voices, including those from LMICs and HICs. The GSRA sought to:

- Identify evidence gaps and highlight priority areas for research that can guide research expenditure and ensure precious resources are spent effectively.
- Assist researchers, funders, practitioners and policymakers with research planning and fundraising.
- Serve as an advocacy tool to signal to stakeholders the areas of research that have been identified as important.
- Serve as a monitoring tool for the field, including monitoring actual research and expenditure against priorities.
- Guide SVRI grant-making.

### Governance structures

The governance structures created for the process was an important part of the GSRA adaptation of CHNRI: instead of one small team alone steering the process [23], the GSRA was developed and governed by three key structures.

The Stewardship Group, comprising key staff and consultants working with the SVRI and EQI, oversaw the overall process, including coordination, design, analysis, reporting and dissemination of the GSRA. The Advisory Group, comprising approximately 30 experts in the VAW prevention and response field, provided expert technical input and advice at key points throughout the process, and were responsible for developing research questions, selecting the criteria for scoring and developing the criteria sub-questions. Care was taken to ensure diversity of representation within the Advisory Group, including members from multiple geographical locations in both LMICs and HICs, Indigenous persons, disability advocates, LGBTQI+ community members, those from different professional backgrounds, including practitioners, researchers and policymakers, and those from diverse age categories including early career researchers. The Global Expert Group, comprising approximately 400 global experts from both LMICs and HICs working on VAW prevention and response, including researchers, practitioners, funders and policymakers, participated in the GSRA process by scoring and ranking priority research questions.

### Six-stage process

**Fig. 1:**
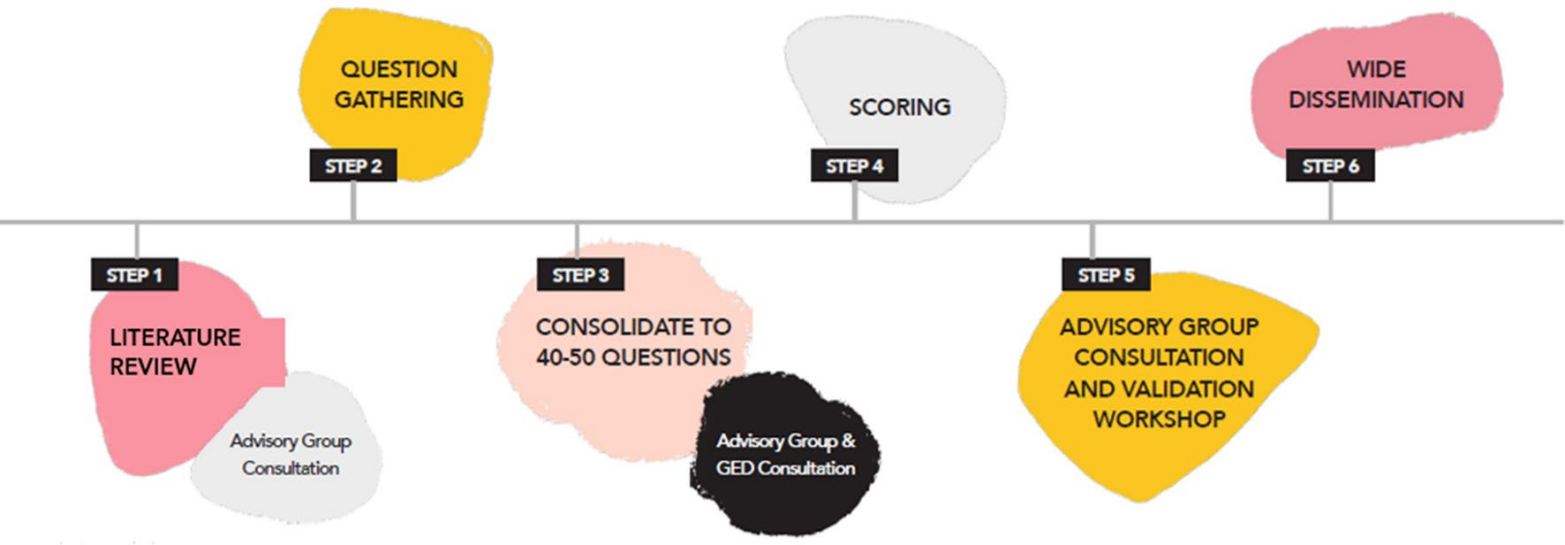
Six stages of the adapted CHNRI approach

The adaptation of the CHNRI method for the GSRA followed six stages, as depicted in Fig. 1.

#### Stage 1: Literature review and development of domains

The first stage of the GSRA comprised a review of the literature, to provide an overview of the key gaps in evidence of VAW in LMICs. Keywords were established for VAW and geographical context and these were combined into a phrase including Boolean terms and applied to title and abstract fields in a number of databases and sources. Due to limitations in the scope of the review and coverage of the literature, several inclusion criteria were used. Papers were included if they: were published in English, French, Mandarin, Portuguese and Spanish; were published from November 2014 to January 2020; were based on studies conducted in LMICs; reported on women’s or adolescent girls’ experience, or men’s or adolescent boys’ perpetration, of IPV, dating violence, NPSV or other forms of sexual violence (e.g., sexual harassment); and treated VAW as a primary theme (not a secondary theme). Literature was included from a range of papers, including systematic reviews, peer-reviewed journal articles and grey literature, and with diverse study designs.

Based on the results of the literature review, four domains were developed to classify the priority research questions, as outlined in Table 1, and these domains were refined in consultation with the Advisory Group.

**Table 1:** Domain definitions

Domain	Definition
1. Research to understand violence against women in its multiple forms	Includes prevalence of different types of VAW, risk and protective factors for VAW experience and perpetration, and the causes and consequences of VAW, including health and psychosocial consequences. Types of VAW include IPV (physical, sexual, emotional and economic IPV, and forms of controlling behaviour) by a current or former partner or spouse; NPSV; sexual harassment and VAW in public and workplace settings; and harmful traditional practices, such as female genital mutilation, so-called ‘honour killings’, and early marriage. This domain also includes new modalities through which violence may occur, including through online and offline technologies and social media
2. Intervention research	Includes research on the development and/or evaluation of any intervention or programme aimed at preventing violence or responding to it, and covers various types of evaluations of interventions, including process, formative and impact evaluations. This domain also includes research that supports the development of theories of change for violence prevention interventions, or research or evaluation conducted to test pathways to change in violence prevention interventions
3. Improving existing interventions	Includes research on existing interventions to understand how positive or promising impacts of interventions can be scaled up to access larger populations, benefit more people and to foster policy and programme development on a more sustainable basis. This could include scale-up research, costing research (i.e., the costs of VAW and of implementing VAW prevention and response interventions), intervention science, process research and other forms of research that generate innovative solutions to improve existing interventions, making them more deliverable, affordable or sustainable. This domain also includes research to understand the impact of developing, implementing and scaling up sustainable violence prevention initiatives at national government level, including policies, frameworks and laws that aim to prevent VAW. A critical element of this domain is access to funding required to adapt and scale up interventions to different contexts, and to ensure that resource distribution is equitable and reaches marginalised groups, including those with intersecting identities
4. Methodological and measurement gaps	Includes new and innovative ways to measure VAW, hierarchies of knowledge, practice- based learning, sticky ethical issues, and monitoring and evaluation of interventions. Methods and measures refer to the methodologies and research instruments we use to measure the different forms of VAW, and their validity, reliability and accuracy. For example, are the measures we use valid (e.g., are they measuring what they are supposed to?) and reliable (e.g., the consistency of how a person answers over time to the same question/scale); are the methods we use (e.g., surveys, questionnaires, scales) scored appropriately; can we use standardised methods and measures across studies; how can we mitigate limitations in measuring accurate VAW prevalence data, including recall bias and social desirability bias? This domain also includes addressing limitations in VAW evaluation approaches, for instance, how to avoid spill-over effects for control or comparison group populations in experimental or quasi-experimental approaches

#### Stage 2: Generation of research questions

After the domains were finalised, the Advisory Group, via an online survey, was asked to develop and submit one research question per domain. Most respondents, however, chose to write multiple questions per domain. The Stewardship Group also completed the question-gathering survey. A total of 34 responses were received, which generated 132 research questions.

#### Stage 3: Consolidation of research questions

The Stewardship Group consolidated the 132 research questions through a three part process: (1) deleting duplicate questions, (2) separating out questions that had different components or multiple potential answers, and (3) reducing the number of questions by applying three criteria (is the question answerable, does the question address a research gap, and is the question relevant?). In relation to the third process, any research question that was unanimously assessed as not meeting the criteria by all members of the Stewardship Group was removed. Through this process, the Stewardship Group was able to reduce the 132 questions down to 57.

The Stewardship Group then facilitated an online workshop with the Advisory Group, in which the 57 research questions were presented. In small working groups, the Advisory Group was asked to discuss any gaps in research questions, and to assess the 57 research questions against the three criteria outlined above, with the aim of reducing the number of research questions to 40. Following the workshop, in line with the recommendations from the Advisory Group, several questions were added and some were rewritten for clarity or to broaden the scope. After several rounds of feedback from the Advisory Group and Stewardship Group, a final list of 41 research questions was created, with 10 questions per domain, except for domain 3 (Improving existing interventions) which had 11 questions.

#### Stage 4: Scoring of research questions

To facilitate the priority-setting exercise, a set of criteria was developed to assess the research questions. To select the criteria, a brief review of the CHNRI criteria was conducted [20, 21, 23, 24] and the ten most common and relevant criteria were posed to the Advisory Group. A survey was distributed to the Advisory Group asking them to rank the ten criteria based on importance to VAW research, with 25 Advisory Group members responding to the survey. The order of the ranking correlated with an individual score (a ranking of 1 would result in a score of 10, 2 in a score of 9 etc.), which produced an overall score for each criterion. The top three criteria were applicability, effectiveness and equity. The Stewardship Group then worked with the Advisory Group to develop and refine definitions and sub-questions for the three criteria. These are presented in Table 2.

**Table 2:** Criteria definitions and sub-questions

Criterion	Definition	Sub-questions
Applicability	Likelihood that the knowledge generated through the proposed research would be implemented in policy and practice and with community involvement	1. Will the research findings produce interventions that are relevant, and applicable to the local context? 2. Will the research findings translate to practical actions and interventions in the next ten years? 3. Will the research benefits balance with the time, costs, resources and community labour required to undertake the research?
Effectiveness	Likelihood that the research will produce novel findings that will generate or improve effective and sustainable interventions	1. Will the research produce novel findings? 2. Will the research contribute to sustainable interventions that can reduce VAW in the long term (e.g., ten years)? 3. Will the proposed research produce findings about good practice that can be effectively communicated and disseminated and where appropriate taken to scale?
Equity	Likelihood that the research findings will lead to interventions that are accessible and equitable to vulnerable groups or, conversely, interventions that will perpetuate inequalities	1. Would you agree the questions would produce findings that would benefit groups with greater vulnerability to violence? 2. Do you think the research question could perpetuate or reinforce inequalities and/or harmful attitudes towards more vulnerable groups? 3. Would the research effectively and meaningfully involve and engage with the affected community?

Two priority setting surveys were developed, one pertaining to the research questions under domains 1 and 2 and the second to research questions under domains 3 and 4. The surveys were distributed in seven languages (English, Spanish, French, Arabic, Mandarin, Hindi and Russian) to experts from the Global Expert Group, Advisory Group and Stewardship Group. This was done in two phases across two separate groups (HIC and LMIC), with domains 1 and 2 sent to the HIC group and domains 3 and 4 sent to the LMIC group in November 2020, and vice versa in December 2020. The decision to develop two separate surveys and disseminate them in a phased approach was made for a number of reasons, including to: reduce survey fatigue; ensure that each domain received some responses; and be able to monitor the number of responses from HICs and LMICs to ensure that the latter were well represented.

Each expert responding to the surveys scored the 41 research options by answering the three sub-questions per criterion listed in Table 2 for each research option. The answers to each question were (a) yes (1 point), (b) no (0 points) or (c) I don’t know (0.5 points). In some instances, experts may not have felt knowledgeable enough to answer a research option and left the option blank. Experts were also asked to provide an additional research question per domain if they felt that any research priorities were not represented in the 41 questions.

The surveys were closed in mid-January 2021 and the results of the surveys were analysed to produce the final GSRA. Research priority scores were calculated by summing all the answers (1, 0.5 or 0), and dividing the sum by the number of answers (blanks were left out). This resulted in a score between 0 and 100%, known as the research priority score (RPS), which represents the extent to which experts believe that the research option best satisfies the priority-setting criteria (applicability, effectiveness or equity).

An average expert agreement (AEA) score was also calculated. The AEA score is a measure of agreement among experts, comprising an average proportion of scorers that agreed on the nine sub-questions asked over the three criteria [23]. In terms of reliability or agreement, an AEA statistic was generated for each research option across the three criteria. The missing (or undecided: 0) responses meant that a Fleiss Kappa statistic to assess agreement was not appropriate [25]. This is in accordance with previous research priority exercises that used the CHNRI methodology [26, 27]. With a large number of scorers and few scoring options, it is possible to create a chance Fleiss Kappa [26]. Although the AEA does not give an indication of statistical significance, it is assumed that funders and or policymakers would find it useful, as it provides a general overview of the agreement between experts [26].

A comparative analysis of scores was carried out, disaggregating the highest and lowest ranked research questions according to survey respondents’ occupation (academics/researchers vs practitioners), gender, setting in which they reside (HIC or LMIC), setting in which they work (HIC or LMIC) and region in which they reside.

#### Stages 5 and 6: Consultation, validation and dissemination

In stage 5, the Stewardship Group hosted an online workshop with the Advisory Group, to present the results of the GSRA to feedback on and validate the findings. Advisory Group members were also invited to provide feedback on the draft GSRA report. In stage 6, the SVRI and EQI launched the GSRA findings to the Global Expert Group and disseminated the results in various formats for different stakeholders, including funders, researchers, practitioners, policymakers and activists.

## Results

### Literature review

A total of 501 papers were included in the literature review: 209 in Asia and the Pacific; 161 in Africa; 41 in Latin America and the Caribbean (LAC); 28 in the Middle East and North Africa (MENA); six in Eastern Europe and Central Asia (EECA); and 56 multi-regional papers.

Thematically, the majority of studies reviewed (*n* = 338) focused on understanding VAW, including the prevalence of or risk factors associated with VAW (mainly IPV, particularly physical and sexual IPV), and the outcomes (including health and psychosocial outcomes) of VAW. There were fewer studies on the impact or effectiveness of VAW interventions (*n* = 148), and very few studies on scale-up and costing of VAW interventions (*n* = 15). Geographically, the literature review found that research in LMICs has been concentrated in certain regions, particularly Africa and South Asia, with much less research conducted in the Pacific, MENA and EECA regions. VAW studies also tend to be concentrated in certain countries within regions, particularly upper-middle income countries (e.g., South Africa and Brazil). Very few VAW studies or interventions specifically targeted women with disabilities (*n* = 2), and there were also few studies (*n* = 8) targeting lesbian, bisexual, transgender, queer and intersex (LBTQI+) populations. Only one paper targeting Indigenous women was identified.

The literature review also identified a large range of methodological and measurement gaps. For example, there is a lack of data on emotional and economic IPV and the majority of studies also report binary measures of IPV, with few reporting frequency, severity or recency of IPV. The review identified few longitudinal studies, and the cross-sectional nature of most studies means that causality and temporality of risk factors cannot be established. Among those studies that did have a longitudinal approach, very few had follow-up data collection 1 year or more after the end of the intervention to be able to accurately assess longevity of change, or to understand additional future outcomes or impacts.

### Research priorities

#### Overview of responses to question gathering survey

A total of 34 responses were received, which generated 132 research questions. The respondents were located in 18 countries, and 56% of respondents (*n* = 19) were from LMICs. Respondents occupied a variety of roles and positions, with most respondents working for non-governmental organisations or universities.

#### Overview of responses to question scoring surveys

There were a total of 214 responses across the two online surveys: 113 responses to the survey covering Domains 1 and 2, and 101 responses to the survey covering Domains 3 and 4. These figures do not necessarily correspond to overall number of participants as some individuals may have completed both surveys while others only one.

Seventy-five percent of respondents identified as female (*n* = 161), and a larger proportion of practitioners (56%, *n* = 120) than researchers (40%, *n* = 86) responded to the survey, with 4% (*n* = 9)—not stating their occupation type. Approximately 60% of respondents across the two surveys (*n* = 128) reported being based in a HIC and 40% (*n* = 86) in an LMIC, with the majority of those in an LMIC residing in a middle-income country and only 11 residing in a low-income country. Out of 214 responses, the largest number came from North America (*n* = 69, predominantly from the USA), followed by western, northern and southern Europe (*n* = 45, almost half from the United Kingdom). Of the 42 responses from Africa, almost half came from respondents residing in South Africa, and 25 responses were received from East Asia and the Pacific (particularly from Australia) and 25 from South Asia (particularly from Bangladesh). Few survey responses were received from the LAC region (*n* = 6) and no responses were received from the EECA and MENA regions.

#### Top five research questions

The results of the scoring process, including the 41 questions listed by overall rank, are included in Table 3. The highest ranked questions in the top five belong to Domain 2 (Intervention research) and Domain 1 (Understanding VAW in its multiple forms).

**Table 3:** Research questions ranked by overall RPS

Overall rank	Research questions	Applicability	Effectiveness	Equity	RPS	AEA	Domain
1.	What types of interventions can effectively prevent multiple forms of violence, and why?	92.4	87.7	72.8	84.3	0.8	Intervention research
2.	What types of interventions are most effective for preventing intimate partner violence (including ‘honour’-based violence) against women facing multiple and intersecting forms of discrimination (including age, poverty, disability, ethnicity, race, sexuality)?	95.1	89.3	63.1	82.5	0.77	Intervention research
3.	How are new feminist social movements (e.g., Me too, Ni una menos) and meninist social movements (Men’s Rights Activists (MRAs), incels etc.) positively or negatively influencing individual, social and policy perspectives related to the experience and perpetration of violence?	89.7	87.9	65.8	81.1	0.76	Understanding VAW in its multiple forms
4.	What interventions work to prevent sexual harassment in institutional settings (in-person or online), including in the workplace and educational settings, and why?	91.2	85.8	62.7	79.9	0.74	Intervention research
5.	What are the impacts (including disability-related impacts) of under-researched forms of IPV on women and girls, including emotional and economic IPV, revenge porn and ‘honour’-based violence?	79.5	82.3	86.2	79.3	0.72	Understanding VAW in its multiple forms
6.	What is the level of intensity needed for social norms change interventions to have sustained impact at the community level, including effectively challenging norms that focus on victim behaviour rather than on the perpetration/choice to use violence?	87.5	86.9	61.9	78.8	0.68	Intervention research
7.	What are the cultural, psychological and economic impacts of colonisation on Indigenous men and women, and how do these impacts influence their behaviours and experiences in respect to VAW?	81.6	82.5	71.2	78.4	0.71	Understanding VAW in its multiple forms
8.	What interventions or elements of interventions are most effective at preventing violence against adolescent girls, and why?	89.7	86.2	58.8	78.2	0.7	Intervention research
9.	What role can formal and informal justice sector reforms, including restorative justice, play in ensuring justice for survivors of violence?	86.5	85.5	62.7	78.2	0.67	Intervention research
10.	Which interventions are most effective at addressing shared risk factors for VAW and VAC in the family environment, leading to a reduction in both types of violence?	84.7	85.6	59.8	76.7	0.66	Intervention research
11.	What are the most effective tools to measure harmful traditional practices against women and girls (including FGM/C, early and forced marriage, crimes committed in the name of honour, dowry-related violence, and son preference)?	82.4	84.7	63.1	76.7	0.64	Methodological and measurement gaps
12.	What methods can be used to measure the intersection and pathways between different types of violence, including polyvictimisation and intersections between VAW and violence against children (VAC)?	88	84.3	57.2	76.5	0.7	Methodological and measurement gaps
13.	How to conduct effective, ethical and inclusive research on VAW using online/virtual/remote methods (including social media) and how should these be adapted to reach marginalised populations?	87.1	86.5	55.1	76.2	0.7	Methodological and measurement gaps
14.	What research methodologies are most appropriate to measure social norm change in violence prevention interventions?	84.8	83.8	55.2	74.6	0.66	Methodological and measurement gaps
15.	In IPV prevention interventions inclusive of women and girls with disabilities, should outcome measures be universal or should some be disability-specific?	81.5	84	57.4	74.3	0.6	Methodological and measurement gaps
16.	How do conflict and fragility exacerbate the multiple forms of violence experienced by women and girls?	79.1	83.8	58.9	73.9	0.6	Understanding VAW in its multiple forms
17.	What alternative modalities (besides in-person programming) are effective in VAW prevention at scale?	87.9	80.7	52.8	73.8	0.67	Improving existing interventions
18.	How can large-scale sector programmes be adapted to optimise their impact on violence prevention and response, particularly education, health, economic development, infrastructure and social protection programmes?	82.7	80.4	57.1	73.4	0.63	Improving existing interventions
19.	How can social movements and feminist activism contribute to preventing and responding to VAW at scale?	84.8	83.4	51.3	73.2	0.66	Improving existing interventions
20.	What are some best practices for ensuring agility and adaptability of VAW interventions, especially those working with marginalised women and girls or operating in complex contexts?	79.6	80.5	58.8	73.0	0.64	Improving existing interventions
21.	What methodologies can be used to measure and attribute the impact of multi-component interventions on VAW prevention, reduction or cessation?	78.9	79.1	60.7	72.9	0.6	Methodological and measurement gaps
22.	What are examples of good practice in addressing recognised ethical challenges of undertaking VAW research in resource-poor settings and/or with marginalised communities?	82.6	81.2	53.2	72.3	0.63	Methodological and measurement gaps
23.	How do we ensure our research impacts policy and programmes and how do we measure that impact?	78	81.9	54.5	71.5	0.56	Methodological and measurement gaps
24.	Do higher costs in resource-intensive violence prevention interventions represent good value for money when taking into account effectiveness in reduction of VAW?	75.4	77.7	60.6	71.2	0.56	Improving existing interventions
25.	How can promising VAW prevention and response interventions from non-emergency settings be adapted to have effect in conflict and humanitarian contexts (e.g., reduced dosage or brevity, different delivery mechanisms)?	75.9	79	57.6	70.8	0.56	Improving existing interventions
26.	In what ways can innovative technologies and interventions be used to detect and prevent online sexual harassment and online intimate partner violence?	85.6	82.9	43.4	70.6	0.65	Intervention research
27.	How can we use tech platforms effectively, safely and cost-efficiently for violence prevention?	85.2	79.5	46.9	70.5	0.62	Improving existing interventions
28.	In what ways can justice institutions be held to account and capacitated to be survivor-centred and hold perpetrators accountable, especially in conflict and post-conflict settings?	76.7	80.9	54.0	70.5	0.56	Improving existing interventions
29.	What are the factors underlying successful intervention and prevention programmes aimed at men, including Indigenous men and other under-researched populations?	77.8	79.1	54.1	70.3	0.55	Intervention research
30.	How do different forms of violence cluster in women and girls with greater vulnerability and what are the characteristics to detect those vulnerable women and girls?	75.2	70.8	60.6	68.9	0.54	Understanding VAW in its multiple forms
31.	What are the causes and drivers of violence against LGBTQI+ women?	75.7	71.3	59.4	68.8	0.55	Understanding VAW in its multiple forms
32.	What are the best methodologies to measure the long-term impacts of violence prevention interventions, including reduction in VAW and other intended and unintended outcomes?	80.5	76.3	49,2	68,7	0.57	Methodological and measurement gaps
33.	What types of interventions are most effective in facilitating gender-transformative change in men and women at scale?	75	79.4	49.6	68.0	0.55	Improving existing interventions
34.	Which analytical approaches (both quantitative and qualitative) are most appropriate for advancing an intersectional approach to research on VAW?	79. 2	76.7	46.0	67.3	0.6	Methodological and measurement gaps
35.	What is the interaction of climate change impacts with the perpetration or experience of VAW?	67.2	73.5	57.9	66.2	0.5	Understanding VAW in its multiple forms
36.	What types of interventions are effective in preventing IPV and other forms of violence against LGBTQ+ people?	77.7	73.7	47.0	66.1	0.56	Intervention research
37.	What is the prevalence of different forms of online and technology-facilitated VAW and what are the risk and protective factors for experience and perpetration of these types of violence?	76.8	75.3	45.9	66.0	0.55	Understanding VAW in its multiple forms
38.	How can police response more adequately address the needs of LGBTQ+ people reporting IPV, non-partner sexual violence and sexual harassment?	74.9	73.2	47.9	65.4	0.54	Improving existing interventions
39.	What steps can be taken to avoid or mitigate resistance to and backlash against women’s rights organisations without compromising the focus and aims of these organisations?	69.3	70.4	54	64.6	0.47	Understanding VAW in its multiple forms
40.	What kinds of faith-based or community-led VAW prevention interventions can be adapted to different faiths, communities and regions effectively?	72.8	67.9	46.2	62.3	0.5	Improving existing interventions
41.	How do social networks act as a protective factor for violence against women and girls?	73	71.7	39.5	61.4	0.54	Understanding VAW in its multiple forms

The highest scoring research question was: What types of interventions can effectively prevent multiple forms of violence, and why? Pertaining to Domain 2 (Intervention research), this question scored highly on the applicability and effectiveness criteria (92.4 and 87.7 out of 100, respectively), and moderately on the equity criteria (72.8 out of 100), and obtained the highest AEA score (0.8).

The second highest scoring research question was: What types of interventions are most effective for preventing intimate partner violence (including ‘honour’-based violence) against women facing multiple and intersecting forms of discrimination (including age, poverty, disability, ethnicity, race, sexuality)? Pertaining to Domain 2 (Intervention research), this question scored very highly on applicability (95.1) and effectiveness (89.3), even higher than for the first ranked question, but the overall RPS (82.5) was pulled down slightly by the equity criteria score (63.1).

The third highest scoring research question belongs to Domain 1 (Understanding VAW in its multiple forms): How are new feminist social movements (eg Me too, Ni una menos) and meninist social movements (Men’s Rights Activists (MRAs), incels etc.) positively or negatively influencing individual, social and policy perspectives related to the experience and perpetration of violence? Scores for the first two criteria were high for this research question (89.7 for applicability and 87.9 for effectiveness), but the lower equity score (65.8) dropped the total RPS (81.1).

The fourth highest scoring research question belongs to Domain 2 (Intervention research): What interventions work to prevent sexual harassment in institutional settings (in-person or online), including in the workplace and educational settings, and why?” Much like the questions outlined above, scores for the first two criteria were high for this research question (91.2 for applicability and 85.8 for effectiveness), but the equity score (62.7) dropped the total RPS (79.9).

The fifth highest scoring research question, from Domain 1 (Understanding VAW in its multiple forms), is: What are the impacts (including disability-related impacts) of under-researched forms of IPV on women and girls, including emotional and economic IPV, revenge porn and ‘honour’-based violence?” This research question had moderately high scores for the applicability and effectiveness criteria (79.5 and 82.3 respectively) and the highest equity score across all questions (86.2). This research question was also the only one in which the equity criterion scored higher than the applicability and effectiveness criteria.

Lower equity scores compared with scores for the other two criteria were observed for almost all research questions, and lower equity scores appear to have driven the placement of research questions in the lower quadrants of the ranking list in Table 3. There are a few possible reasons for low equity scores, leading to a reduced RPS for those questions ranked at the bottom overall. Questions with the lowest equity scores were more likely to be specific, including referring to specific populations (e.g., LBTQI+ people, adolescent girls), specific types or modalities of violence (e.g., online sexual harassment, technology-facilitated VAW), specific types of interventions (e.g., those targeting faith-based actors) or a specific risk or protective factor for violence (e.g., social networks). Conversely, research questions with the highest equity scores tended to be broader in scope and more generalised, including coverage of wider populations (e.g., women facing multiple and intersecting forms of discrimination), multiple forms of violence (e.g., under-researched forms of IPV) or interventions that target multiple forms of violence. Feedback provided by survey respondents also suggested that they struggled with scoring research questions according to the equity criterion as they felt that it was not possible to determine from the question itself whether research would be equitable, potentially leading to more conservative scoring.

#### Variations according to expert characteristics

There was strong consistency in top ranked research questions according to experts’ occupation, with four out of the top five research questions ranked overall being ranked in the top five by both VAW practitioners and researchers. However, there were also some variations. Among practitioners, there is a preference for questions related to Intervention research, with four out of five top questions belonging to Domain 2. In contrast, researchers’ top five questions included two from Domain 2 (Intervention research), two from Domain 1 (Understanding VAW in its multiple forms), and one question related to Methodology and measurement gaps (Domain 4), which ranked third for this group.

There was much more variation in ranking of research questions according to the gender of experts. While the top five questions for female experts were the same as the top five ranked overall, only two questions preferred by male experts fell within the top five rank overall. Furthermore, there was variation in the types of domains corresponding to the top five questions, with all questions in male experts’ top five belonging to Domain 2 (Intervention research), including those questions ranked as 2nd, 4th, 6th, 8th and 9th overall (see Table 3).

There was also strong consistency in the ranking of research questions according to experts’ geographical characteristics, albeit with some exceptions. For instance, the fifth ranked question overall on the impacts of under-researched forms of IPV (including disability-related impacts) did not score in the top five for experts living and working in LMICs. Instead, the question related to effective interventions preventing violence against adolescent girls was ranked 5th among those living and working in LMICs despite being ranked 8th overall. There were also some variations in the top five questions among experts based in or working in HICs. The fourth ranked question overall, on interventions that prevent sexual harassment in institutional settings, did not score in the top five questions for experts based in or working in HICs. Fifth ranked questions for those based in or working in HICs were, respectively, related to tools to measure harmful traditional practices against women and girls (ranked 11th overall) and the impacts of colonisation on women and men (ranked 7th overall).

There were a number of notable regional variations in the ranking of research questions. For example, the Domain 1 question on feminist and meninist social movements (ranked third overall), did not score in the top five questions for experts in East and South-East Asia and the Pacific. Further, the fourth ranked question overall, on preventing sexual harassment in institutional settings, did not score in the top five for experts based in Europe. While the fifth ranked question overall on the impacts (including disability impacts) of under-researched forms of IPV was scored in the top five among experts based in East and South-East Asia and the Pacific and North America, it appears to be less of a priority in other regional settings.

## Discussion

The GSRA findings suggest that currently the VAW field is shifting towards intervention research. Extensive research has been conducted over the last two decades to understand the prevalence, drivers and consequences of violence against women and girls, much of this led by the WHO. This has resulted in strong global and regional estimates [9], so it is understandable that there is perhaps less prioritisation of research on prevalence and drivers etc.

A shift in the field towards intervention research does not necessarily mean that understanding VAW in its multiple forms is not important. The literature review identified several population gaps, including few studies addressing violence against women in vulnerable groups, particularly violence against women with disabilities and LGBTQI+ people. It is clear from the research questions identified in the priority setting exercise that there is strong interest in understanding violence against populations that have been overlooked in the past, with two research questions in the top five explicitly referencing women with disabilities, or women facing multiple and intersecting forms of discrimination.

The literature review also identified that most studies focused predominantly on IPV, particularly physical or sexual IPV. It is notable that in the priority-setting exercise, among the top five questions overall, one referred to multiple forms of violence, one to under-researched forms of IPV (e.g., emotional and economic IPV) and another to sexual harassment, suggesting that there is an increasing recognition in the field of the need to expand evidence to different types of VAW.

The literature review identified very little research conducted in low-income countries, and the priority setting exercise included the voices of fewer experts from low-income countries compared with middle-income countries. Hence, it is possible that research on prevalence, drivers and consequences is still very much needed in low-income countries. However, a logical next step in LMIC countries that have generated extensive evidence on understanding VAW in its multiple forms is to better understand what interventions work to respond to and prevent VAW.

The lack of prioritisation of research on improving existing interventions may be due to a larger emphasis on developing and understanding the impact of different types of interventions, although a natural progression in future would be to build evidence on how successful interventions can be scaled up. The lack of prioritisation of studies or publications on methodologies and measures may reflect that the field is more squarely focused on programming to end VAW at this point in time. It may also reflect the larger number of practitioners than researchers/academics participating in the priority-setting exercise.

Despite consistency in the overall scoring of questions, when disaggregated by experts’ personal and geographical characteristics, there were some notable differences. While questions related to intervention research appear to be a particularly strong priority for practitioners, they appear to be less so for researchers. It is to be expected that practitioners would be particularly interested in research to understand the effectiveness of programmes.

There were also some notable differences in research priorities between LMICs and HICs, and across regions. For example, a question related to interventions aimed at preventing violence against adolescent girls was ranked fifth overall for experts based in and working in LMICs; however, this question was not ranked in the top five by experts from HICs. We know that VAW and VAC overlap during adolescence, as some forms of violence are often first experienced during this period, or become elevated due to an individual’s age [28]. This may be particularly relevant in LMICs, where early marriage is more common and therefore an important priority for those from the region [29]. The voices of those based in LMICs should be elevated when determining research priorities for those settings, as they are more likely to understand the local needs and lived realities [17, 18].

### Challenges and limitations

There were a number of challenges and limitations in developing the GSRA. The literature review conducted at the start of the process was not a systematic or exhaustive review of the state of the evidence on VAW but rather a literature review to extract key trends in evidence gaps across LMIC regions and to help the Stewardship Group refine the research Domains.

There were also some limitations in the processes associated with scoring research questions. Despite efforts to make the process as accessible as possible, some respondents found the priority-setting surveys confusing and reported that they would have preferred to rank or vote on the research questions rather than applying the criteria through the sub-questions, which was reported to have been time-intensive. Some respondents also found it difficult to answer the sub-questions as they were highly context-dependent, particularly in the case of the equity criterion. Another limitation in the scoring process is the relatively small number of people who responded to the surveys (214) compared with the number of members of the Global Expert Group and Advisory Group who were invited to respond (approximately 430). This may be due to a number of reasons, including the length and complexity of the survey, and the process taking place during the Covid-19 pandemic which may have restricted some people’s access to internet. Nevertheless, the response rate was high when compared with other research agenda-setting processes drawing from the CHNRI method [30].

Finally, because the Global Expert Group was initially composed from the networks of the Stewardship Group and the Advisory Group, it was not as broad as it could have been. For example, most respondents undertook the survey in English, with four surveys completed in French and five surveys completed in Spanish, which suggests that the findings are still heavily skewed towards English-speaking stakeholders. Furthermore, although the GSRA focuses on VAW research in LMICs, respondents were predominantly based in HICs, with the MENA and EECA regions particularly unrepresented.

Despite these limitations, diverse stakeholders from across the globe fed into the development of the GSRA at various stages of the process and the findings present important insights into priorities for the VAW prevention and response field, highlighting avenues for future research that are priority-driven and provide empirical guidance for interventions, programmes, policies and advocacy.

## Conclusion

Bringing together researchers, practitioners, activists, funders and decisions makers to co-create a GSRA has revealed that there is substantial expert agreement on the priority research questions for the VAW prevention and response field in LMICs in the next five years, although with some differences across regions and expert characteristics. Nevertheless, there were some limitations in the process that have led to important learnings about research priority setting. While the GSRA’s adaptation of the CHNRI method increased the diversity of voices, a number of voices were still excluded, particularly from certain regions. Future processes need to improve stakeholder engagement and modalities for engagement to ensure they are genuinely supporting the decolonisation of knowledge and centering of diverse voices in setting research priorities. These processes should also be evaluated in order to ensure that the field is continually learning about what is working and not working in research priority setting.

## Data Availability

Dataset available from corresponding author on reasonable request.

## Abbreviations

CHNRI: Child Health and Nutrition Research Initiative
EECA: Eastern Europe and Central Asia
EQI: Equality Institute
GSRA: Global Shared Research Agenda
IPV: Intimate partner violence
HIC: High income country
LAC: Latin America and the Caribbean
LMIC: Low- or middle-income country
MENA: Middle East and North Africa
NPSV: Non-partner sexual violence
SVRI: Sexual Violence Research Initiative
VAW: Violence against women
